# Basic tips for pregnant women during the COVID-19 pandemic

**DOI:** 10.18332/ejm/125723

**Published:** 2020-09-04

**Authors:** Ioanna Papari

**Affiliations:** 1 National Public Health Organization PHILOS, Athens, Greece

**Keywords:** pandemic, COVID-19, pregnancy, antenatal care

Dear Editor,

Any data that we have had so far concerning the transmission of COVID-19 during pregnancy are limited. According to the existing data, pregnant women do not seem to be more vulnerable to the virus than any other members of the population. However, pregnant women who suffer from other diseases, at the same time, run a higher risk to suffer from the virus than anyone else with the same diseases^1^.

We emphasize that in case a pregnant woman has a cough, temperature, and respiratory distress, she should stay home and should not be transferred to any medical care facility unless telephone contact has been made with either a doctor or a midwife, so that she will avoid standing in crowded queues. Taking into consideration any other possible reasons for the symptoms she has during pregnancy as well as in the case of a deterioration of the symptoms, a pregnant woman should get in touch with a doctor or a midwife. In any case, a pregnant woman whether she is considered an unconfirmed or confirmed case should follow her doctor’s or midwife’s guidelines so as to ensure both her own and her baby’s good health condition^2-5^.

In order to protect themselves, it is advisable that pregnant women should^4^:

- Follow the guidelines provided by authorities^3^
- Get informed about COVID-19 from reliable sources^3^
- Remain physically active so that they can ensure their physical healthcare^3^
- Contact their midwife or doctor to acquire guidelines concerning any possible changes in their scheduled appointments as well as their delivery plan, including the women who have suffered from COVID-19^3,5,6^
- Have been supplied with the basic medicine and medical equipment to relieve temperature. In case they take any prescribed medicine, they should have an adequate stock of it^3,5^
- Remain socially active. They should meet their family, their friends and medical staff and they should be prepared in case COVID-19 spreads in their area or in case they suffered^3^
- Take thorough care of their personal sanitary condition^3,5,6^.

Research is still in progress and the data are continuously changing and being enriched. The global medical society is working consistently so that an effective medicine can be found to treat the symptoms and a vaccine is found so that, globally, humanity will manage to return to normal life as soon as possible^7^.

## References

[1] American College of Obstetricians and Gynecologists Novel Coronavirus 2019 (COVID-19).

[2] Du L, Gu YB, Cui MQ, Li WX, Wang J, Zhu LP, Xu B (2020). Investigation on demands for antenatal care services among 2 002 pregnant women during the epidemic of COVID-19 in Shanghai.

[3] European Centre for Disease Prevention and Control Know, prepare, proteck: Information on Covid-19 for pregnant women.

[4] Vivilaki VG, Asimaki E (2020). Respectful midwifery care during the COVID-19 pandemic. Eur J Midwifery.

[5] Omer S, Ali S, Babar ZUD (2020). Preventive measures and management of COVID-19 in pregnancy. Drugs Ther Perspect.

[6] Royal College of Obstetricians & Gynecologists Coronavirus inflection and pregnancy: Information for pregnant women and their families. https://www.rcog.org.uk/en/guidelines-research-services/guidelines/coronavirus-pregnancy/covid-19-virus-infection-and-pregnancy.

[7] Li H, Liu SM, Yu XH, Tang SL, Tang CK (2020). Coronavirus disease 2019 (COVID-19): current status and future perspectives. Int J Antimicrob Agents.

